# Human IRES Atlas: an integrative platform for studying IRES-driven translational regulation in humans

**DOI:** 10.1093/database/baab025

**Published:** 2021-05-18

**Authors:** Tzu-Hsien Yang, Chung-Yu Wang, Hsiu-Chun Tsai, Cheng-Tse Liu

**Affiliations:** Department of Information Management, National University of Kaohsiung, 700, Kaohsiung University Rd., Nanzih District, Kaohsiung 811, Taiwan; Department of Information Management, National University of Kaohsiung, 700, Kaohsiung University Rd., Nanzih District, Kaohsiung 811, Taiwan; Department of Information Management, National University of Kaohsiung, 700, Kaohsiung University Rd., Nanzih District, Kaohsiung 811, Taiwan; Department of Information Management, National University of Kaohsiung, 700, Kaohsiung University Rd., Nanzih District, Kaohsiung 811, Taiwan

## Abstract

It is now known that cap-independent translation initiation facilitated by internal ribosome entry sites (IRESs) is vital in selective cellular protein synthesis under stress and different physiological conditions. However, three problems make it hard to understand transcriptome-wide cellular IRES-mediated translation initiation mechanisms: (i) complex interplay between IRESs and other translation initiation–related information, (ii) reliability issue of *in silico* cellular IRES investigation and (iii) labor-intensive *in vivo* IRES identification. In this research, we constructed the Human IRES Atlas database for a comprehensive understanding of cellular IRESs in humans. First, currently available and suitable IRES prediction tools (IRESfinder, PatSearch and IRESpy) were used to obtain transcriptome-wide human IRESs. Then, we collected eight genres of translation initiation–related features to help study the potential molecular mechanisms of each of the putative IRESs. Three functional tests (conservation, structural RNA–protein scores and conditional translation efficiency) were devised to evaluate the functionality of the identified putative IRESs. Moreover, an easy-to-use interface and an IRES–translation initiation interaction map for each gene transcript were implemented to help understand the interactions between IRESs and translation initiation–related features. Researchers can easily search/browse an IRES of interest using the web interface and deduce testable mechanism hypotheses of human IRES-driven translation initiation based on the integrated results. In summary, Human IRES Atlas integrates putative IRES elements and translation initiation–related experiments for better usage of these data and deduction of mechanism hypotheses.

**Database URL**: http://cobishss0.im.nuk.edu.tw/Human_IRES_Atlas/

## Background

Cells in proliferation adapt their metabolism to environmental stimuli through the regulation of protein synthesis, and translational regulation can provide instant protein level adjustment for handling sudden environmental changes and stress responses (1). Importantly, translation initiation is widely considered to be a vital step in translational regulation (2). For eukaryotes, the complex phase of cellular translation initiation can be categorized into two different types: cap-dependent translation initiation and cap-independent translation initiation (3). Under normal situations, eukaryotic canonical cap-dependent translation initiation is started by the pre-initiation complex formed with initiators tRNA, eIF2, eIF3 and eIF4 to scan for the start codon. However, when facing stressed environments or other physiological challenges, cellular translation can be initiated in a cap-independent manner via the help of the internal ribosome entry site (IRES) elements. IRES-mediated translational initiation was first discovered in poliovirus (4). Since then, scientists have found different IRES elements in other viruses (5) and in cellular genes (6). Cellular IRES elements are now known to be closely related to selective cellular protein synthesis under stress and physiopathological conditions (7) and can have profound effects on human tumor cells (8). Therefore, it is crucial to investigate cellular IRES elements, thus unraveling the nature of cellular stress responses and tumor therapeutic targets.

Currently known cellular IRES elements are mostly found in genes involved in stress, mitosis or apoptosis (9). They usually bare sequence or structural motifs that can recruit the translation machinery to initiate the translation initiation stage (10). Although cellular IRESs generally show lower structural similarities to one another and are less structured than viral IRESs, they can be roughly categorized into three different functional genres with some identifiable patterns (11). First of all, IRES structural forms within 5ʹ untranslated regions (UTRs) can interact with RNA-binding proteins known as IRES *trans*-acting factors (ITAFs) to remodel their structures, thus opening the hindrance to ribosome recruitment. Second, upstream open reading frames (uORFs) in the neighborhood of an IRES can retain ribosomes in the uORF regions to transform the IRES structure into an active form using amino acid stress. The resulting active conformed IRES then helps presume the cap-independent translation initiation. Third, IRES can modulate different regulation signals around 5ʹ UTR and recruit internal ribosomes accordingly. Some known translational regulation signals such as cap inhibition, uORF translation, ITAFs or RNA G-quadruplexes can trigger the bypassing of canonical cap-dependent translation initiation. These basic known cellular IRES functions provide clues to select the transcriptome-wide IRES sequences in humans.

Various translation initiation–related cellular features are associated with the aforementioned cellular IRES functions. They should be simultaneously considered to elucidate IRES-mediated translational regulation mechanisms. According to the known cellular IRES functions, IRESs reveal complicated interplay with the data of uORFs around IRES sequences, ITAF-binding targets, the IRES structural forms, conservation of the functional sequences and the TIS triplets around IRESs. Therefore, to understand IRES-mediated gene regulation and deduce testable molecular mechanisms, there is an urgent need to articulate the interconnections between IRES elements and these translation initiation–related features. While many of these translation regulation features have been measured and studied, most of the resources are still fragmentary over diverse literature or supplementary repositories. Some of the valuable experimental results require tedious data pre-processing to be used in a comparative analysis. Furthermore, current bicistronic reporter assays for discovering novel IRES elements require series of experiments to rule out the noises from cryptic promoters or spicing activities (12), leading to the time-consuming, small-scale and labor-intensive nature of these experimental approaches. Systematic identification of IRES elements currently resorts mainly to *in silico* methods to first narrow down the search space. As a consequence, studying IRES in humans requires the following two advances: (i) evaluate the functionality of the *in silico* predicted putative IRESs (13, 14) and (ii) integrate IRES elements and translation initiation–related information to elucidate and propose testable regulatory mechanisms for each identified IRES.

The complicated connections between IRES elements and translation initiation–related features bring about an immediate demand to construct an integrative IRES database. This database should perform a systematic transcriptome-wide IRES identification and integrate the comprehensive translational regulatory features to help biologists convey researches on IRES-driven translational regulation. In this research, we constructed a database called Human IRES Atlas to accomplish these goals. Human IRES Atlas is designed for easier understanding and evaluating the functionality of IRES candidates. It aims to reduce the long computing time of large-scale IRES screening on the human transcriptome and integrate the translation-related information for better hypothesis generation and evaluation. We first performed comprehensive putative IRES searching on the human transcriptome using currently available IRES prediction tools. To assess the functionality of the putative IRES, we considered their sequence conservation, structural RNA–protein interaction (RPI) scores and conditional translation efficiency. For elucidating the complex connections between IRES elements and translation initiation–related features, we integrated eight types of datasets that probe vital translation initiation information to help biologists infer possible IRES-mediated translational regulatory mechanisms. For analyzing the interconnections between IRES and translation initiation–related features for individual gene transcripts, an easy-to-use interface and interaction maps were constructed. Human IRES Atlas provides the functionality to search for an IRES of interest or browse the human transcriptome for putative IRES lists satisfying the user-customized filtering criteria. In summary, this database is designed in an integrative manner for simultaneous consideration of putative IRESs and translation initiation features. Many critical observations and testable mechanism hypotheses can be retrieved from this integration. Human IRES Atlas is available online at http://cobishss0.im.nuk.edu.tw/Human_IRES_Atlas/.

## Construction and contents

### Construction of Human IRES Atlas

**Figure 1.**
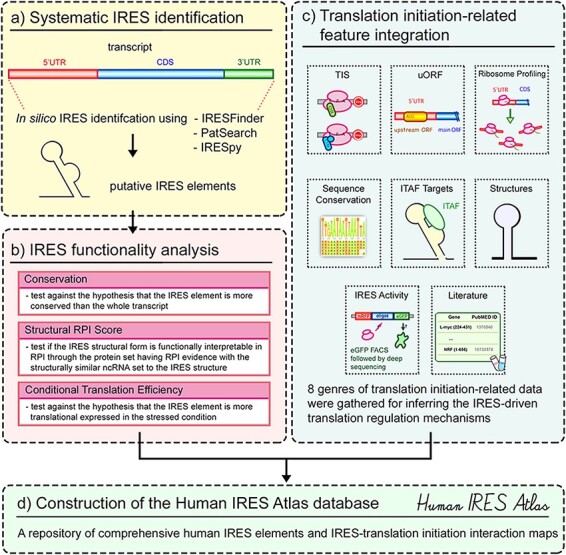
Overview of the Human IRES Atlas database.

Human IRES Atlas deposits systematically identified putative IRES elements and integrates comprehensive translation initiation–related features to assist the study of IRES-driven translational regulation in humans. The overall database contents and construction can be roughly divided into four parts (see Figure 1). First, the transcriptome-wide putative human IRES screening was performed (Figure 1a). Currently available and suitable IRES prediction tools were used to identify the putative IRES elements in the human transcriptome. To evaluate their potential as functional IRESs, three functionality tests were conducted for each of the putative cellular IRES elements: sequence conservation (among different mammalian species), structural RPI scores and conditional translation efficiency (Figure 1b). Users can assess the functionality of the putative IRES for the queried gene based on these computed False Discovery Rate (FDR)-corrected test *P*-values. Third, eight genres of translation initiation–related datasets were collected and integrated into the database to facilitate biologists to form testable mechanism hypotheses for IRES-driven translational regulation (Figure 1c). In the final stage of database construction, an easy-to-use interface to query and browse the potential IRESs with the associated translation initiation features was constructed (Figure 1d). A transcript detail page that presents the complicated connections between IRES elements and translation initiation features can be found for the selected transcript. Testable functional hypotheses of the putative IRESs identified within the transcript can be inferred from the IRES structure conformation and other probed translation initiation–related features in the detail page. Overall, Human IRES Atlas aims to deposit transcriptome-wide putative human IRESs and articulate the complicated interplay between IRESs and translation initiation features. This integration helps suggest testable molecular mechanism hypotheses for subsequent IRES-mediated translation regulation experiments.

The construction of Human IRES Atlas can be divided into four different parts. First, the systematic transcriptome-wide IRES identification on all human transcripts was performed based on the evaluated screening tools (part a). The IRES functionality from sequence conservation, structural RPI score and conditional translation efficiency was then estimated for each identified putative IRES (part b). To further provide potential translational regulation information, eight types of datasets were collected and integrated into the repository to assist the deduction of IRES-mediated translational regulation mechanism hypotheses (part c). Finally, an easy-to-use query interface and a transcript detail page with translation initiation interaction maps were implemented to help biologists infer testable hypotheses for subsequent IRES-driven translation initiation research experiments (part d).

### Transcriptome-wide human IRES screening

**Figure 2.**
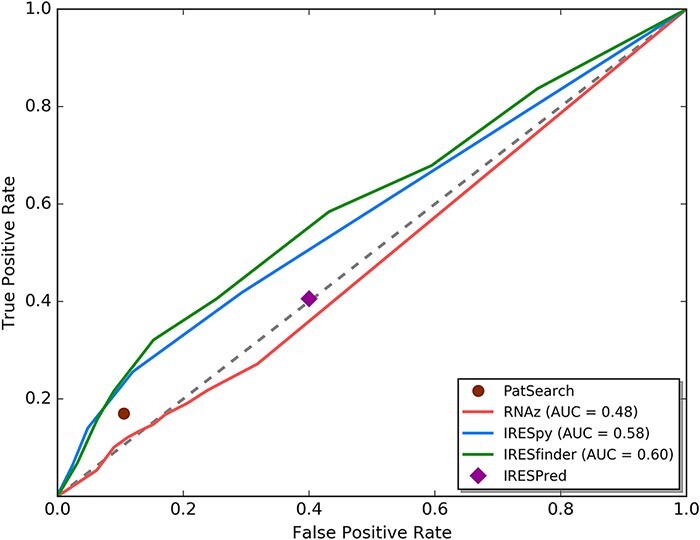
ROC curve analysis for currently available IRES prediction tools on 5ʹ UTRs.

We first performed transcriptome-wide IRES element identification on all human 5ʹ UTRs. The human 5ʹ UTR sequences were downloaded from the UCSC Genome Browser database (15) (Dec. 2013 assembly of the human genome, hg38). 53 971 messenger RNA (mRNA) transcripts for 19 367 genes were available in total. The sequences before the coding sequence (CDS) regions of each of these transcripts were defined as the 5ʹ UTR sequences. Based on the defined 5ʹ UTR sequences, currently available computational IRES scanning algorithms were evaluated to be used in this research. Five IRES prediction tools [IRESfinder (16), PatSearch (17), RNAz (18), IRESPred (19) and IRESpy (20)] are still currently available. To evaluate these tools, we collected a ground truth dataset with verified IRES sequences as the positive set and random sequences as the negative set. The positive set of 190 known IRES elements is obtained from IRESbase (21) and the review work of Baird *et al.* (22). Since it is known that house-keeping genes depend mainly on cap-dependent translation initiation (23), we randomly sampled 190 sequences from the 5ʹ UTRs of the house-keeping genes annotated by Eisenberg and Levanon (24) to be the negative set. We forced the negative set to be of the same sequence length distribution as the positive set. Using the receiver operating characteristic curve technique (see Figure 2), we can see that RNAz and IRESPred have performance similar to random guessing, and IRESfinder, IRESpy and PatSearch have area under curve larger than 0.5. Based on this analysis, only IRESpy, PatSearch and IRESfinder are suitable for obtaining potential IRES elements in 5ʹ UTRs. Therefore, IRESpy, PatSearch and IRESfinder were applied in the screening of possible IRESs in 5ʹ UTRs. The thresholds used in the database construction are 0.7 and 0.9 for IRESpy and IRESfinder, respectively.

We used the ROC curve analysis technique to evaluate the results of different IRES prediction tools. From the ROC curve, RNAz and IRESPred have performance similar to random guessing. IRESfinder, IRESpy and PatSearch have AUC larger than 0.5. Based on this analysis, only IRESpy, PatSearch and IRESfinder are suitable for obtaining potential IRES elements in 5ʹ UTRs.

Currently, most computational IRES identification tools assumed input sequences to be of medium lengths (<174 bps) in their model training process (25). Hence, in this research, we applied the sliding window approach proposed by Zhao *et al.* (16) to systematically identify transcriptome-wide IRES elements. We first divide the 5ʹ UTR sequence into segments of 174 nts long with a step size of 10 nts between consecutive overlapping segments. Each of these fragments was then fed separately into different IRES identification tools to calculate the probability of the sequence to have a potential functional IRES element. In the last stage of the IRES scanning process, consecutive potential IRES elements identified by the same IRES prediction tool were concatenated to form the final functional IRES elements. 17 689 IRES sequence segments were identified in human 5ʹ UTRs using these three IRES screening tools.

It is known that the false discovery rate of the prediction process can be reduced if a potential IRES sequence is identified by more than one IRES screening tool (13). Hence to decrease the false positives in IRES screening, we adopted the nested minimal sequence definition to cross-reference the identified IRESs from different screening tools. For a group of nested potential IRES elements, we classified these IRESs into nested sets and took the minimal sequences in the nested sets as the representative IRES sequence segments. Among the 17 689 IRES elements identified by the three IRES screening tools, 17 013 minimal IRES elements in 5ʹ UTRs were cross-referenced and deposited in Human IRES Atlas.

Although most well-known cellular IRESs were found in the 5ʹ UTRs, some IRESs in CDSs and 3ʹ UTRs were also reported to drive the translation of novel proteins or protein isoforms (26–28). IRES screening experiments revealed that 3ʹ UTRs also contained many IRESs (12). Therefore, we have also extended the Human IRES Atlas database to include the potential IRES elements in CDSs (including the main ORF start codon) and 3ʹ UTRs of mRNAs. In the IRES identification beyond 5ʹ UTRs, only IRESpy was used. For PatSearch and IRESfinder, the designed features and the training process were performed on only 5ʹ UTR sequences. Thus, these two tools are not suitable for the IRES identification process on regions other than 5ʹ UTRs. Since no existing tool was trained on Long non-coding RNA (lncRNAs), it is currently unavailable to predict IRESs on lncRNAs and will be carried out when further advances are made in model construction. Extra 1868 IRESs on the CDSs/3ʹ UTRs were included. In total, there are 18 881 IRESs deposited in Human IRES Atlas.

### Data collection and pre-processing of translation initiation feature data

Eight genres of translation initiation feature data were gathered and integrated into the database to assist biologists in understanding the interplay between IRES elements and translation initiation. The integration also helps users propose testable molecular mechanism hypotheses for IRES-driven translational regulation. The following contents were collected and integrated in this repository: translation initiation site (TIS) probing experiments, uORF sequences, sequence conservation scores (among mammalian species), ribosome profiling data, structure information of the IRES elements, experimentally identified ITAF target sequences, IRES activity bicistronic measurements and literature-curated IRES verification experiment evidence. Details of the deposited datasets are described in the following subsections.

In Human IRES Atlas, the putative human IRES elements were systematically identified by computational methods. To form reliable IRES-driven translation initiation mechanism hypotheses, we are required to understand the functionality of these putative IRES identifications. Recent studies on cellular IRES have revealed that known human IRES elements are rich in their GC contents to form some special ITAF interacting sequences or structures (11, 22). However, there are still some IRESs that are characterized by their high AU-rich contents (29). These AU-rich IRES elements interact with specific ITAF chaperones that shape the RNA structure into forms that facilitate translation initiation (30). Functional sequences in the genome are usually believed to be conserved among different species (31, 32). Based on the understanding of cellular IRES elements, three functionality tests are also performed with the collected data in this database: conservation, structural RPI scores and conditional translation efficiency between stressed and normal states. Note that the thresholds of the functional tests should be treated as statistical type I error control for the functional aspects. It should not be misinterpreted as the prediction confidence since it is reported that some human IRESs are not conserved in mammals or lack complete structural forms (30).

#### Data of sequence conservation between mammalian species

Functional elements are often evolutionary conserved among different closely related species (31, 32). Hence when the specified sequences are to carry out vital cellular functions, they tend to be significantly more conserved among different related species (33). The base-by-base sequence conservation level can be captured by the phastCons conservation scores (34). The phastCons conservation scores were based on multiple alignments of human and other 19 mammalian genomes (baboon, bushbaby, bonobo, chimp, crab-eating macaque, gibbon, golden snub-nosed monkey, gorilla, green monkey, marmoset, mouse lemur, orangutan, proboscis monkey, rhesus, squirrel monkey, tarsier, tree shrew, mouse and dog). We mapped the conservation scores in the genome to transcripts using the transcript mapping annotated by the UCSC Genome Annotation database (15). To test the conservation significance of a putative IRES element, we used the rank-sum test (35) to calculate the *P*-value against the hypothesis that an IRES element of interest is more conserved than its corresponding transcript in phastCons conservation scores. Multiple hypotheses testing bias was corrected by the FDR control approach for the calculated *P*-values among all IRES elements.

#### Ribosome profiling data and conditional translation efficiency

Translation initiation in eukaryotes proceeds in a series of complicated steps that lead to the final ribosome complex formation. Hence, the ribosome-binding sites located within the 5ʹ UTR sequences under different cellular conditions can reveal important translational regulation hints for active translation programs (36). The experimental procedure called ribosome profiling (ribo-seq) can help identify the transcriptome-wide in vivo ribosome existence and can be used to indicate whether the given sequences show higher translation efficiency in the stress condition. To help users obtain the conditional translation efficiency for a given transcript, we collected the ribosome profiling data of tunicamycin-treated (SRX870808 and SRX870809) and untreated (SRX870804 and SRX870805) HEK293T cells (37). Tunicamycin is a toxin that blocks N-linked glycosylation of Endoplasmic Reticulum (ER)-residing proteins and induces cell depth, leading to ER stress. ER stress induces translational reprogramming that favors alternative translation such as IRES mechanisms. From these ribo-seq experiment results, users can recognize if the transcript is differentially expressed between normal and stress conditions. By simultaneously considering the ITAF data and the ribo-seq results, users can postulate if the stress response of the transcript is mainly regulated by IRES-mediated (38), uORF-mediated (39) or IRES–uORF-interplay (40) translation initiation. To integrate the ribosome profiling results, we first used the NCBI SRA Toolkit to download the sequencing results and then transformed the results into the fastq format. We followed the peak calling procedure used in the HRPDviewer (41) to obtain the transcript nucleotide-level reads for both conditions. First, the adaptor linker sequences or poly-(A) tails of the short reads are trimmed by Cutadapt (42). These trimmed reads are then mapped to the hg38 reference transcriptome using RSEM (43) with the bowtie engine. Finally, the reads per million mapped reads (RPM) values are calculated on all transcript nucleotide positions. By performing the rank-sum test on the RPM values between the stressed and normal conditions, users can get the idea if the identified IRES is stress-induced. Multiple hypotheses testing bias was corrected by the FDR control approach for the calculated *P*-values among all IRES elements.

#### RPI scores and structural information within IRESs

Structural features have been found in several well-studied cellular IRESs (44, 45). Although the structure–function relationship seems to be less rigid in cellular IRESs than in the viral ones, current investigation of known cellular IRESs has indicated that at least some structural modules play important roles with other ITAFs in cellular IRES-mediated translation initiation (11, 30). Hence, studying the potential regulatory IRES structures can help infer the potential functional molecular mechanisms. 12 currently available RNA secondary structure prediction tools [RNAFold (46), RNALfold (47), RNAprob (48), MaxExpect (49), RNAalifold (50), TurboFold (51), SPARSE (52), MXSCARNA (53), aliFreeFold (54), LocaRNA (55), comRNA (56) and RME (57)] were used to comprehensively identify the candidate secondary structures of the putative IRES elements. We further implemented an RPI score to estimate the given IRES’s tendency to interact with proteins. This score is derived from a comparison between the queried sequence and verified RPIs curated in the RNAInter database (58).

Structurally similar non-coding RNA (ncRNA) usually carry out similar roles in RNA–protein complexes. Thus, proteins showing interactions with them tend to work in similar biological processes. For estimating the tendency of a given RNA candidate structure to interact with a protein, we resorted to the human ncRNAs having verified RPIs curated in the RNAInter database (58). Specifically, by BLASTing these ncRNAs to the BRAlibaseII structure benchmark dataset (59), we collected a reference set with known structures and their corresponding interacting protein set. To obtain the potential IRES structural forms, the abovementioned 12 different RNA structure prediction tools were integrated in this research to produce candidate structures for a given IRES sequence. For each candidate structure, an ncRNA set consisting of the top 10 structurally similar ncRNAs from the constructed reference set was extracted. The similarity between a candidate structure and a reference ncRNA structure was measured by the general RNA structure distance defined by Jiang *et al.* (60). The defined general RNA structure distance considers both sequence mismatches and structure pairing mismatches. From the extracted structurally similar ncRNA set of a candidate structure, proteins interacting with these structurally similar ncRNAs were listed based on the data from the RNAInter database. On the listed protein set, we performed the protein interaction prevalence test and the ontology enrichment test as defined in previous researches (61). In brief, the protein interaction prevalence test evaluates if the protein set forms an enriched protein complex, and the ontology enrichment test checks if the protein set has statistically significant annotated functional ontology terms. The enrichment *P*-values are defined as the RPI scores. A given IRES’s candidate structure with the lowest RPI score among its structure predictions was selected as its representative IRES structure. Lower RPI scores of the predicted structure indicate that the queried sequence is structurally similar to ncRNAs selected from the reference RNA collection and is more likely to interact with a protein complex that regulates a particular cellular function(s). The RPI scores among all putative IRESs in Human IRES Atlas were corrected by the FDR multiple hypothesis control approach.

#### Data of ITAF-binding events

Cellular IRESs in eukaryotes are reported to cooperate with specialized RNA-binding protein called ITAFs in the regulation of the ribosome translation machinery (9, 23). By acting as molecular chaperones or RNA structure remodelers, ITAFs identify particular sequences or structures in IRES elements to help facilitate the translation initiation factor recruitment and initiation complex formation (38). Hence, investigating the connections between ITAF-binding sites and IRES locations can help understand the potential cellular translational regulation mechanisms. Various cellular ITAFs have been reported in humans. Some of their binding targets have been experimentally probed through the transcriptome-wide cross-linking and immunoprecipitation (CLIP) or the photoactivatable-ribonucleoside enhanced cross-linking and immunoprecipitation (PAR-CLIP) techniques at a nucleotide-level resolution. We collected the CLIP and PAR-CLIP-binding events for the following ITAFs from the doRiNA database (62) and remapped them into the hg38 transcriptome: 4 001 271 FUS-binding sites, 4899 hnRNPC-binding sites, 30 593 hnRNPL-binding sites, 621 116 HuR/ELAVL1-binding sites, 806 p54nrb-binding sites and 1 367 825 eIF4A-3-binding sites. Simultaneously considering the predicted IRES elements, the identified ITAF-binding events and the potential IRES structural forms can help users infer possible IRES-mediated translational regulation molecular mechanisms.

#### Data of translation initiation site probing data

The translation machinery is completely formed to start polypeptide chain synthesis on special triplets called TISs. Canonical translation initiation uses the AUG triplet as the normal start codon. However, many non-AUG triplets have been shown to provide alternative starting sites for initiating uORF translation or the IRES-driven initiation (63). Hence, the TIS signals around or within IRES elements can provide translation initiation evidence that supports the regulatory effects of these IRES elements. Two different types of TIS data were collected and mapped to human transcript 5ʹ UTRs. The first type that indicates translation initiation is the TIS peaks probed by sequencing techniques. The high-throughput transcriptome-wide TIS positioning for humans is collected from the global translation initiation sequencing (GTI-seq) performed in the work by Lee *et al*. (64). In their work, two different ribosome E-site translation inhibitors, namely the cycloheximide (CHX) and lactimidomycin (LTM), were used in HEK293 cells. CHX reports elongating ribosomes by blocking translation elongation and LTM probes initiating ribosomes since it preferentially binds to the TIS, while the elongating ribosomes run off the mRNAs. We followed the same analysis procedure used by TISDB (65) to map their sequencing results to the human hg38 transcriptome. In brief, the GTI-seq data were first mapped to the hg38 genome and transcriptome by Tophat (66). The 13th nucleotide of a uniquely mapped read was identified as the start codon recognized in translation initiation. The initiation sites with statistically significant read counts were then determined using the zero-truncated binomial negative model. In total, 11 395 TIS peaks were identified in the 5ʹ UTRs of the transcripts. These experimentally probed TISs are called experimentally probed translation initiation site (eTIS) in this database. The eTIS experimental contexts suggest either uORF start codons resulting from cap-dependent scanning or cap-independent IRES activity under growth conditions in these particular cells. The second group of translation initiation data is the non-canonical start codon predicted by the PreTIS software (67). PreTIS constructed from a linear regression approach was trained on different ribosome profiling datasets, significant Position Weight Matrix (PWM) and biologically motivated features. Using the PreTIS tool, the potential non-canonical TISs were generated. We adopted a confidence level of 0.9 in this research to obtain 13 900 predicted non-canonical TIS within the human 5ʹ UTR sequences. The collected non-canonical TIS is called nTIS in this database.

#### Data of uORF sequences

Researchers have reported that the stalling of ribosomes in the uORF regions can regulate IRES-driven translation initiation (68). uORF translations can mediate 5ʹ UTR structural remodeling to conform the regulated IRES element into the active form (69) or unfold the IRES structure to repress IRES activity (40). Hence, considering the connections between IRES elements and uORFs within 5ʹ UTRs could help indicate the potential regulatory mechanisms of IRESs. We adopted the analysis procedure proposed by Ye *et al*. (70) to obtain the uORFs in each of the transcripts. uORFs were identified by scanning the 5ʹ UTRs of the transcripts to obtain the sequences that start with the start codon AUG and continue with a three-nucleotide reading frame until the stop codon is encountered. In total, 131 612 uORFs starting from human 5ʹ UTRs were collected in this database.

#### Evidence of IRES activity

Recently, Weingarten-Gabbay *et al*. (12) devised a systematic bicistronic assay coupled with fluorescence-activated cell sorting to uncover the human transcript sequences with cap-independent translation activity. In their work, they measured the enhanced green fluorescent protein (eGFP) expression scores as the IRES-mediated translation activity in the prepared oligo pEF1 vector library consisting of the virus genome, human genome and reported IRES mutations. They performed the bicistronic experiments for 6946 5ʹ UTRs of 5058 genes in humans, about one-fourth of all human genes. These IRES activity values can indicate the existence of IRES elements for the given sequence segments. Following the experimental design settings of Weingarten-Gabbay *et al*., the IRES activity was designated as background expression when the oligos were not detected in adjacent eGFP bins during GFP sorting but had >100 reads in the full mRFP-oligos-eGFP library, indicating these background oligos did not convey cap-independent translation signals in the experiments. The oligos were assigned ‘NaN’ if they got <100 reads in the full monomeric red fluorescent protein (mRFP)-oligos-eGFP library, representing a lack of identification confidence. Other normal real numbers represent the extent of IRES activity of the regulatory sequences. When values larger than the background signal are reported for a given identified IRES, subsequent verification experiments to rule out cryptic promoters and splicing activity can be designed to confirm the existence of IRES functionality. In total, we collected and mapped the probed human oligo sequences with IRES activity measurement to 39 700 transcript segments (E-value <1E-6, identity percentage >85%, query cover >85%, promoter activity <0.2 and splicing activity >−2.5) in this database.

#### Collection of literature-curated IRESs

Since the first discovery of the viral IRES sequence in poliovirus, various researchers have studied and described diverse cellular IRES elements in the literature. These results have been collected in various review papers. Some databases were also constructed to deposit IRES verification experiments. However, all of these databases either stopped updating the contents or were no longer available to the public. In this research, we collected those literature-verified human IRESs from the IRESite database (71), the IRESbase database (21) and the review works of Baird *et al*. (22), Leppek *et al*. (11) and Stoneley *et al*. (30). These verified IRES sequences were mapped back to the transcripts of the reported IRES-regulating genes by BLASTN (E-value <1E-6, identity percentage >85% and query cover >85%). In total, 659 IRESs with experimental evidence were retrieved from IRESite, IRESbase and the literature.

### Implementation of the Human IRES Atlas web interface

IRES identification and the translation initiation features data pre-processing were carried out using Python. The web interface of the database is constructed using the PHP CodeIgniter MVC framework. IRES data and the related datasets were deposited through MySQL.

## Utility and discussion

### Database interface

In Human IRES Atlas, four essential database functions are implemented to help select IRES elements of interest: (i) search function: users can search the putative IRESs for different transcripts of a given gene. (ii) Filter and browse function: users are allowed to browse the deposited transcriptome-wide human IRESs categorized by chromosomes or KEGG pathway annotations of genes regulated by them. The KEGG pathway annotation helps users identify the IRES elements that regulate genes involved in the same biological pathway. This criterion helps biologists check if a particular biological pathway consists of different potential IRES regulation mechanisms. To enable users to select only the putative IRESs with significant *q*-values for the three functional tests, we have further implemented the *q*-value filters in the browse page. Users can set the intended *q*-value threshold(s) for the functional test(s), and the database will show only the potential IRES elements with significant *q*-values. The resulting IRES list can be downloaded in the browse page. (iii) Transcript detail page: after selecting the transcript of interest in the query results, a detailed translation mechanism deduction page is provided. In this page, the detailed IRES summary and translation initiation–related features are integrated to help understand and infer the testable mechanism hypotheses for each IRES. (iv) Download function: the integrative IRES–translation initiation interaction map and the supplemented experimental datasets can be downloaded as plain text files for subsequent analyses. An easy-to-use web interface was built to facilitate these functions.

**Figure 3.**
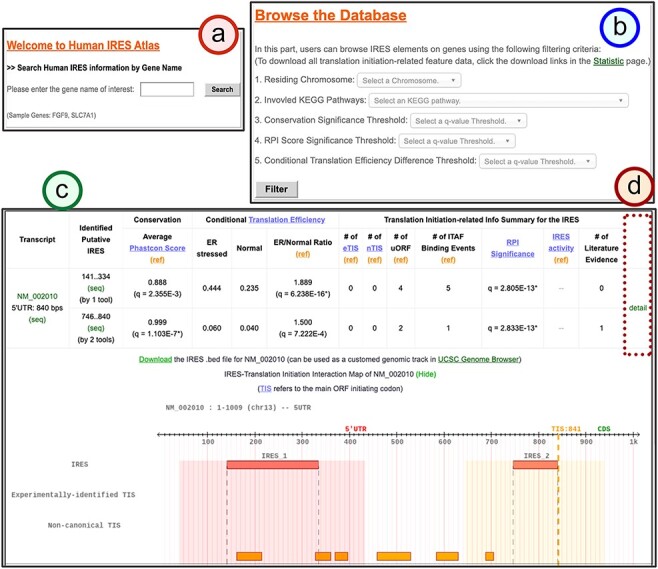
The search result page for the identified IRESs of a given gene.

Users can type in a particular gene name or its aliases in the search page (Figure 3a). Users can also browse the database to find IRESs categorized by chromosomes and/or KEGG pathway annotations of genes regulated by them (Figure 3b). The putative IRESs and the summary of the corresponding translation initiation–related information are then presented in a tabular form (Figure 3c). For each transcript of the input gene, Human IRES Atlas summaries the brief transcript information, the putative IRES elements found on the transcripts, the number of prediction tools that specify this sequence to be a potential IRES, the functionality test results for each identified IRES element and finally the summaries of other translation initiation–related data observed within IRES regions. Three tests (conservation significance, RPI score significance and conditional translation efficiency significance) are provided to assess the functionality of each identified IRES. The summary of the translation initiation–related data matched to an IRES region includes the following information: (i) the number of eTIS (experimentally probed TIS) found in the upstream to the downstream 100 bps of the IRES element, (ii) the number of nTIS (non-canonical TIS) predicted in the upstream to the downstream 100 bps of the IRES element, (iii) the number of uORF observed in the upstream and downstream 100 bps of the IRES element, (iv) the number of ITAF-binding events that overlap and interact with the IRES element, (v) IRES activity measured for the IRES element and (vi) the number of literature IRES evidence that overlaps with the IRES prediction. Some IRES elements have been shown to use the *cis* uORFs and TISs to mediate their activity (45, 69). The amino acid stress or the stalling ribosome near an IRES can trigger a structural switch of the IRES to an active conformation, leading to IRES-mediated translation initiation. Therefore, we included extra 100 bps upstream and downstream of an IRES when associating TISs and uORFs to it. For ITAFs, they usually bind and remodel IRES structures to set up the ribosome-binding platform (38).

(a) The search mode: users can type in the gene of interest to search for the identified IRESs on the given gene transcripts. (b) The browse mode: users can filter out the IRESs categorized by their residing chromosomes, annotated KEGG pathways of the regulated genes and the thresholds of IRES functionality *q*-values. (c) The result table for the identified IRES elements in all transcript isoforms of the queried gene. In this table, three IRES functionality test results and other translational regulatory features for each identified IRES element are summarized. (d) The link to the detail page of the selected transcript.

Below the summary table of the translation initiation–related features, an interaction map between translation initiation–related features for the transcript can be viewed in this table for better data visualization (Figure 3c). For each transcript, one can click the ‘detail’ hyperlink (Figure 3d) to obtain the detail page that lists the complete information of the putative IRES elements, the visualized IRES–translation initiation interaction maps and the translational regulation information at a single nucleotide resolution. Both the genomic chromosomal coordinates and the transcript locations for different translation initiation–related information are listed in the detail page. Moreover, we have also provided the *.bed* files for the putative IRES track and different transcription-related information tracks. These files can facilitate users to integrate the application with the genome browser. In the last part, the implemented transcript detail page assists in forming molecular mechanism hypotheses for IRES-driven translation initiation. The walkthrough of the detail page is provided along with the case study.

### Case study

**Figure 4.**
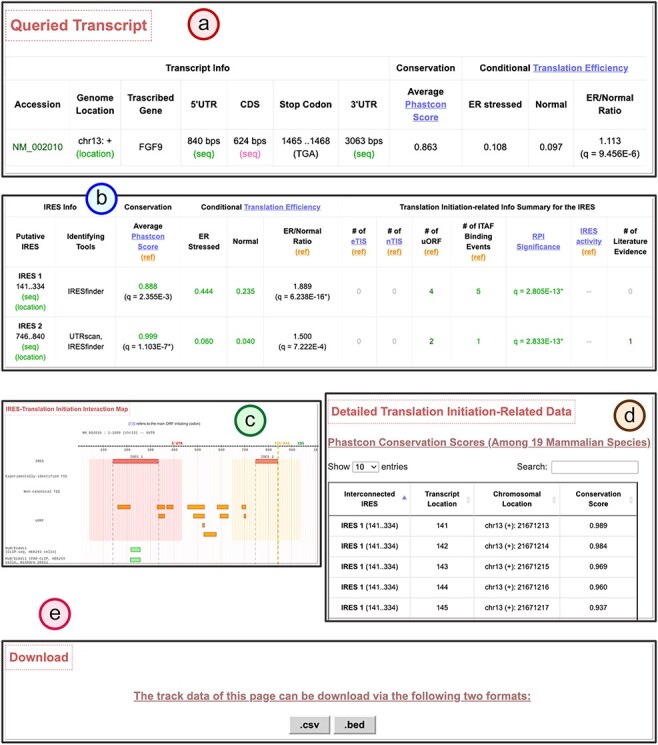
The transcript detail page showing the potential IRES-driven translation regulatory program of the given transcript.

Human IRES Atlas aims to integrate the transcriptome-wide human IRESs and translational regulatory features. The integration provides ways to infer testable mechanism hypotheses for IRES-mediated translation regulation. One can use the IRES–translation initiation interaction map and the queried transcript’s detailed information to deduce such hypotheses. We discuss the IRES-mediated regulatory program of human FGF9 in detail to demonstrate the biological applicability of the constructed database. Human Fibroblast Growth Factor 9 (FGF9) is the gene that transcribes and translates to a protein that acts as an autocrine/paracrine growth factor in lung branching morphogenesis (72), bone development (73) and even cancer progression (74). It has been reported that the FGF9 mRNA is expressed in embryos while the FGF9 protein is identified to be of low level in most cells and is restrictedly expressed in specific cell organs (75). Hence, it is of interest to biologists that how the FGF9 transcripts are translationally regulated in human cells. In Human IRES Atlas, the FGF9 transcript map of IRES–translation initiation interactions provides clues for deducing the testable mechanism hypotheses. There is an identified overlapping IRES region (IRES2: 746.840) that was found by at least two IRES screening tools and is calculated to be conserved and significant in functional RPI scores (*q* = 2.83E-13*) (Figure 4a and b). In the FGF9 IRES–translation initiation interaction map, very low levels of TIS and ribosome occupancy signals were found (Figure 4c), suggesting the low translation level in normal conditions. Further, uORFs were discovered in proximity to these structurally significant IRES elements. Finally, HuR-binding signals were observed to be overlapped with the putative IRES. Details of the translation initiation–related data found around each IRES element are listed in the fourth part of the page (Figure 4d). A link to download the text files in the *.csv* format that contain the full information of the detail page is provided for users (Figure 4e).

(a) The brief information table of the queried gene transcript. (b) The translation initiation–related feature summary table for the identified IRES elements within the queried gene transcript. (c) The IRES–translation initiation interaction map for the queried gene transcript. (d) The detail of the translation initiation–related feature data found for each IRES. (e) The download function to obtain the *.csv* or *.bed* formats of all information in the detail page.

**Figure 5.**
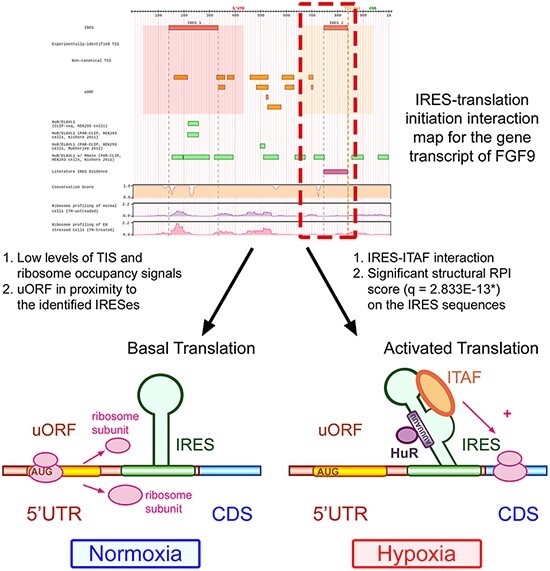
Human IRES Atlas helps suggest testable IRES-mediated translational regulation mechanism hypotheses.

By careful consideration of the integrated information, we can deduce that FGF9 transcripts could be translationally regulated through the interplay between the structural IRES, uORF segments and ITAFs (see Figure 5). By the effort of molecular biologists, it is now verified that there is indeed a functional IRES structure near the end of the 5ʹ UTR of the FGF9 transcript. The uORF segments found around the IRES elements were shown to maintain the low protein expression level (40). The IRES structure induced under hypoxia recruits the ribosome to the CDS and activates the FGF9 protein synthesis with IRES–ITAF interactions. Although it is later found that the ITAF HuR may bind to the AU-rich elements in FGF9 transcripts (76), further studies and experiments should be carried out to figure out whether other ITAFs interact with these IRES elements. These findings conform to the hypotheses suggested by the constructed database. In conclusion, translation regulatory programs can be inferred with the facilitation of Human IRES Atlas.

For the FGF9 5ʹ UTR, the IRES–translation initiation interaction map suggests that (1) the transcript is of low translation level and (2) some regulatory program between the RPI interpretable IRESs, the nearby uORFs and some ITAFs may exist. From experimental literature evidence, we know that the uORF represses FGF9 protein synthesis in normal conditions. Some ITAFs bind to the IRES to start cap-independent translation under hypoxia, verifying the testable hypotheses suggested by the constructed database.

### Comparison with related works

Human IRES Atlas was created for two primary purposes: (i) to provide comprehensive and systematic IRES identifications in the human species and (ii) to integrate IRES elements and translation initiation–related information for biologists to understand the interconnections between them. By constructing a comprehensive IRES–translation initiation interaction map, biologists can get testable hypotheses on cellular IRES elements and devise subsequent verification experiments. Currently, four existing databases and datasets are devoted to similar tasks as Human IRES Atlas and thus are compared in the following discussion.

Two IRES databases [IRESite (71) and IRESbase (21)] were designed to curate and deposit IRES verification experiments from the literature. Researchers have carried out different bicistronic experiments for studying IRES activity of certain genes. Nevertheless, most of the information is fragmentary in different literature papers. Therefore, these two databases tried to gather and store these valuable records. While these two works collected valuable IRES experimental data, till now there are still few cellular IRES elements that have been experimentally verified and deposited. Only 26 human mRNA transcripts were evaluated to contain IRES elements in IRESite and 112 human IRESs were collected in IRESbase. Potential IRES-driven translational regulation programs in the human transcriptome cannot be easily deduced through these two databases due to the lack of an integrative platform for understanding the interactions between IRES elements and the related translation initiation features. Since there are only few available verified IRES elements in the literature, there is still a need to carry out systematic *in silico* IRES identification and construct a comprehensive IRES–translation initiation interaction map to complement the sparsity of IRES verification experiments.

Another related work called IRES Omnibus (77) studied human IRESs through systematic IRES prediction. In the preliminary work of IRES Omnibus, Chen and Tseng set up an in-house IRES prediction system based on the PatSearch tool to screen through human 5ʹ UTR sequences for potential IRES elements. In their work, IREZone, the special regions consisting of contiguous overlapping RNA structures, and IRES elements were predicted and collected. Using the IRES and IREZone repository, novel sequence patterns and IRES-related cellular functions were investigated. However, in their preliminary identification, merely one IRES prediction tool was used, and the complicated connections between IRES elements and translation initiation–related features were ignored. Hence, the constructed preliminary human IRES map may suffer from high false positives. Further, it would be beneficial for biologists to deduce the potential IRES-related regulatory molecular mechanisms if different high throughput translation initiation–related datasets can be integrated into a user-friendly platform.

The study by Weingarten-Gabbay *et al.* (12) is another touchstone in the field. We then evaluated the IRES identification sensitivity and specificity for Human IRES Atlas and the work of Weingarten-Gabbay *et al.* on a ground truth IRES dataset consisting of verified IRES sequences as the positive set and random sequences as the negative set. The positive set of 190 known IRES elements is obtained from the IRESbase (21) and the review work of Baird *et al.* (22). Since it is known that house-keeping genes depend mainly on cap-dependent translation initiation (23), we randomly sampled 190 sequences from the 5ʹ UTRs of annotated house-keeping genes (24) to be the negative set. An identified IRES in a dataset is defined to be a true positive if *>*20% of the base pairs overlap with some sequence in the positive set. A sequence from the negative set is called a true negative for one identification method if no identified sequence has *>*20% overlapping base pairs with it. On this ground truth IRES set, the results of Weingarten-Gabbay *et al.* obtain a sensitivity of 0.19 and a false-positive rate (FPR) of 0.037. The putative IRESs deposited in Human IRES Atlas have a sensitivity of 0.32 and an FPR of 0.226 on the ground truth dataset. We can see from these results that Human IRES Atlas achieves a better sensitivity with a small sacrifice of the FPR. Furthermore, if we merge the potential IRESs in Human IRES Atlas and the results of Weingarten-Gabbay *et al.*, a sensitivity of 0.43 and an FPR of 0.258 are observed. The near linearly independent sensitivity gain means that the two results are mostly complementary. By examining the data of Weingarten-Gabbay *et al.*, 19% of the literature-curated IRESs were found. Thirty-two percent of the literature-curated IRESs could be determined in Human IRES Atlas. While oversampling 5ʹ UTRs can lead to higher chances of overlapping with the true positive set, it also increases the possibility of overlapping the putative IRESs with the true negative set, raising the FPR. There is a compromise between the sensitivity and the FPR when oversampling 5ʹ UTRs. The putative IRES elements deposited in Human IRES Atlas are estimated to have a higher sensitivity than the corresponding FPR. Hence, Human IRES Atlas provides extra insights for IRES-mediated translation regulation in humans.

Human IRES Atlas is the first database explicitly designed to overcome current obstacles in studying human IRES elements. It also fulfills the necessity for deducing testable molecular mechanism hypotheses. In Human IRES Atlas, three different IRES prediction tools (IRESFinder, PatSearch and IRESpy) were used for systematic IRES screening. The results of different tools were collected by selecting the nested consensus minimal sequences among different prediction algorithms. Hence the prediction uncertainty and false discovery rate inherent in different tools were minimized during the identification process. To further help evaluate the functionality of the putative IRESs, three tests (conservation, structural RPI scores and conditional translation efficiency) were designed and performed. Besides performing the transcriptome-wide human IRES identification, we also integrated eight categories of translation initiation–related datasets. Biologists can further understand the IRES-driven translation regulation and develop testable molecular mechanism hypotheses for subsequent research through the translation initiation–related features. In summary, Human IRES Atlas integrates more data and provides more functionality than existing tools. It specifically aims to perform systematic IRES identification and help deduce the IRES-driven cellular translation regulation mechanisms.

### Issues related to the constructed database

The Human IRES Atlas database is constructed by depositing systematically identified *in silico* IRESs and various translation initiation–related data. Based on the eight gathered categories of translation initiation–related features, we can assess the significance of the putative IRESs and deduce potential IRES-driven translation regulation mechanism hypotheses. However, several issues may exist when interpreting the results generated from the systems-wide data integration process. Researchers usually perturbed cells and conducted distinct high-throughput experiments for gathering genome-wide information (78). However, different high-throughput experiments may be performed under different environmental conditions, cellular states or cell types. This experimental discrepancy causes inevitable systematic bias in the data integration process (79). Hence, some detailed subtleties should be considered when designing subsequent verification experiments based on the hypotheses formed from the systems view of data integration.

Diverse statistical analysis strategies were applied to the high-throughput experiment datasets. For example, we adopted the eTIS data by thresholding the positional read-count fold change and some other minimum detection requirement between samples treated with the ribosome E-site translation inhibitors CHX and LTM (64). The high-throughput screening of IRES activity adopted in this database was probed using fluorescence measurements (12). Different statistical analysis strategies provide different false discovery rates. In general, noises resulted from the data statistical analysis were assumed to be orthogonal in different high-throughput experimental results (35). Hence, by simultaneously combining experimental data of similar targeted systems, the false positive issue can be reduced. In Human IRES Atlas, we have gathered diverse translation initiation–related data and used different screening tools in the systematic putative IRES identification. This cross-referencing screening reduces the impact of false positives. For instance, TISs based on CHX/LTM experimental analysis and PreTIS prediction were both collected to lessen the false positive impact in TIS location information. We will keep Human IRES Atlas up-to-date with newly published datasets when more data or methods are released.

Data integration in multicellular organisms often brings about the issue of cellular systems bias. In metazoa species, correct temporal and spatial gene expression of the same genome is the core control on cell differentiation (13). While different combinations of cell types in data integration may provide distinct false positives, the multicellular systems problem should be taken care of in using the constructed database. IRES activity data were probed *in vitro* based on experiments on sequence libraries. Non-canonical TIS and uORF information were identified *in silico* by investigating comparative genomics and sequence contents. These data reveal the normal status of the translation program. To assist in cell type-specific or condition-specific IRES verification experimental design, one can refer to ribosome profiling experiments conducted under different conditions/cell types or refer to special cellular conditions where the ITAF-binding events were observed. Diverse ribosome profiling sequencing results are fragmentary in the literature. Recently, a database called HPRDviewer (41) is constructed to deposit comprehensive human ribosome profiling datasets. In Human IRES Atlas, the conditional ribosome profiling results performed under the stressed and normal conditions are shown. More condition-specific human ribosome profiling information can be found in HPRDviewer. Through consulting more detailed ribosome profiling experiments or the ITAF-binding event conditions, one can further minimize the impact of cell type bias inherent in the data integration process.

## Conclusions

In this research, a novel database called Human IRES Atlas was constructed to deposit the systematic *in silico* identified IRESs from the human transcriptome. Besides the transcriptome-wide putative IRES identification, eight genres of translation initiation–related datasets were integrated and three novel IRES functionality tests were designed. To facilitate the understanding of the connections between IRES elements and these translation initiation–related features, an easy-to-use interface for the integrated results was implemented for biologists to infer possible molecular mechanisms of human IRES-driven translation initiation. Researchers can easily search the IRES of interest or browse the IRESs on genes involved in specific biological pathways with the database’s user-friendly web-interface. By simultaneously studying the human IRES and the translation initiation–related features, the database can provide testable molecular mechanism hypotheses on human gene translation regulation. We believe that Human IRES Atlas can help biologists better study mechanisms of cellular IRES-driven translation initiation, IRES-related cancer progression and other IRES-related genetic disorders.
